# Cutlery

**Published:** 1848-01

**Authors:** 


### CUTLERY.

It is rather remarkable, that no Cutler has located in our midst, where we have two Medical Schools, a numerous Medical and Dental Faculty, with a population of about 65,000 people, and increasing with unexampled rapidity. In our judgment, there is no place in the United States, which offers the same desirable inducements to a young man of ability, and steady industrious habits.—St. L. Ed.

